# Aloe vera jelly dessert supplemented with *Lactobacillus curvatus* encapsulated in *Plantago major* mucilage and sodium alginate: Characterization of physicochemical, sensory properties and survivability against low pH, salt, heat, and cold storage

**DOI:** 10.1002/fsn3.4003

**Published:** 2024-02-13

**Authors:** Seyed Saeed Sekhavatizadeh, Maryam Derakhshan, Mohammad Ganje, Saeid Hosseinzadeh

**Affiliations:** ^1^ Fars Agricultural and Natural Resources Research and Education Center AREEO Shiraz Iran; ^2^ Department of Food Science and Technology Bushehr Institute of Kherad Higher Education Bushehr Iran; ^3^ Department of Agriculture, Minab Higher Education Center University of Hormozgan Bandar Abbas Iran; ^4^ Department of Food Hygiene and Public Health, School of Veterinary Medicine Shiraz University Shiraz Iran

**Keywords:** extrusion, gastrointestinal, heat stress, *Lactobacillus curvatus*, salt acid condition

## Abstract

The goal of this research was to assess the free *Lactobacillus curvatus* (FLC) and microencapsulated *L. curvatus* (MLC) survivability using sodium alginate and *Plantago major* mucilage (PMM), as a second layer to produce probiotic aloe vera jelly dessert (AVJD). To determine bead characteristics, the aspect ratio of the bead, survival in 72°C, and cold storage were assessed as well as for AVJD, survivability of probiotics in simulated gastrointestinal condition (SGIC), and storage time. The results showed that all the beads are spherical (aspect ratio = 1.12), and under heat stress conditions, MLC showed a higher survival rate (50.15%) compared to FLC (not detected after 5 min). The number of survived probiotics in the MLC sample (8.65 log CFU/mL) was higher than FLC (7.52 log CFU/g) on the 28th day. In AVJD, the MLC survived at a minimum scientific adequate number of probiotics (6.88 log CFU/mL) on the 28th day. In SGIC, the final survival rates of FLC and MLC samples were 14.24% and 71.04%, respectively. These results suggest that using alginate and PMM is a promising method to protect *L. curvatus (LC)* from harsh environmental conditions and in AVJD.

## Graphical abstract

PMM is proposed to use in Lactobacillus curvatus microcapsules for enhancing the heat stress. The MLC count was higher than 7 log CFU/g in the 28th storage time. During simulation GI condition, the survival of MLC was higher than FLC, in AVJD.

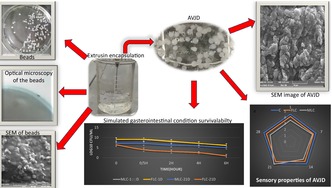

## 1 INTRODUCTION

The International Scientific Association on Prebiotics and Probiotics defined probiotics as “live microorganisms that, when administered in adequate amounts, confer a health benefit on the host” (Hill et al., 2014). A dose of greater than 10^6^ Colony‐Forming Units (CFU) per g or mL was proposed as a proper dose of viable cells for probiotic products (Terpou et al., 2019). These health benefits include prevention of intestinal inflammation, immunomodulatory properties, stimulation of cell regeneration and mucus production in the gut, reduction of diarrheal side effects, and protection against pathogenic bacteria (Bustos et al., 2023). *Bifidobacteria* and *Lactic acid bacteria*, comprising several strains of *Lactobacillus*, *Streptococcus*, and *Enterococcus*, make up the majority of probiotics (Shrivastava & Bhatu, 2023). Some types of *lactic acid bacteria* are considered probiotics and can be found in various food products, such as *Lactobacillus acidophilus, Lactobacillus rhamnosus, Lactobacillus bulgaricus, Lactobacillus casei*, and *Lactobacillus reuteri* (Yu et al., 2023). The LC is essential for health and food applications. The LC is part of the microbiota of many fermented products and is characterized by bacteriogenic activity (Funck et al., 2019).

The survival of probiotics is influenced by numerous factors during the processing and storage of foods. These factors relate to unique product properties such as processing‐related treatments, salt presence, sugar content, water activity, colors, titratable acidity, artificial flavors, bacteriocins, pH, hydrogen peroxide, and oxygen. They may also be related to the following factors: conditions such as refrigeration, storage, heat treatments, incubation temperatures, production scale, packaging materials, fermentation conditions, microbiological properties such as inoculation rates and proportions, and microbial strains (Terpou et al., 2019).

Survival after passage through the gastrointestinal tract, viability during food processing, storage, and host health benefits are essential for selecting probiotics (Pourjafar et al., 2020). The harsh condition of the stomach is not tolerated by most bacteria. Thus, the selection of the exact probiotic strain is a crucial element in formulating a healthy food product (El‐Sohaimy & Hussain, 2023).

Microencapsulation technology applies to preserve the viability of probiotics even under adverse conditions and to protect probiotics by creating a physical barrier from the environment. The microencapsulated spheres containing the core material are in the micro‐ to millimeter size range. Various microencapsulation methods such as emulsion, spray drying, and extrusion exist (Kowsalya et al., 2023). One of the other techniques used in microencapsulation is extrusion. Microencapsulation by extrusion is advantageous. It is nontoxic, efficient to handle, and economical (Amin et al., 2021). We hypothesized that microencapsulation could improve LC viability during storage and under harsh conditions.

Microencapsulation is an immobilization technique that encapsulates cells placed in a matrix of encapsulating material. Functionality, stability, type of release, encapsulated concentration, cost carrier biodegradable materials biocompatible, food‐grade, and capable of barrier formation are the most important criteria to select the encapsulation materials (Nami et al., 2017). Encapsulating materials commonly used in microencapsulation techniques include sodium alginate, chitosan, gellan, xanthan gum, whey protein, starch, carrageenan, Arabic gum, cellulose, and pectin (Parsana et al., 2023). Among them, sodium alginate, the most commonly used, is insoluble at acidic pH; thus, it protects the living cells of probiotics from gastric acid as they pass through the stomach, swells in alkaline pH conditions, and releases probiotic cells in the intestine (Kowalska et al., 2022).

Using a second layer as an additional coating of alginate beads addresses these issues. The second layer may improve beads in harsh conditions (Karimi et al., 2021). For instance, the porosity of alginate beads during gastric transit is considerably reduced by applying chitosan‐coated alginate beads to finally provide a more stable physicochemical appearance (Dokoohaki et al., 2019). One of the materials that can be used in microencapsulation is seed mucilage. The mucilage could be used as a second layer to improve sodium alginate function. *Plantago Major* is a large genus of the *Plantago* family, comprising more than 250 species, widely distributed geographically in temperate and high‐altitude tropical regions. It is a perennial herb with a fibrous root system, a rosette of elliptical leaves, and multiple long inflorescences (Lyu et al., 2023). The seed contains polysaccharides that are comprised arabinose, xylose, rhamnose, galactose, glucose, glucuronic acid, and galacturonic acid and can be used as active natural polymers for developing edible coatings (Noshad et al., 2021). We hypothesized that *Plantago major* mucilage (PMM) could be used on the bead's surface as a second layer.

The role of desserts in the menu becomes very crucial. Besides being refreshing, aloe vera jelly also has health benefits. Aloe vera contains fats, proteins, and carbohydrates that produce energy. The presence of vitamins A and C in aloe vera makes it an appropriate dessert for eye health and maintaining immunity. In contrast, other dessert products are generally sweet but often contain slight functional advantages (Wachyuni et al., 2020). So far, no studies have been conducted on the production of probiotic AVJD. Therefore, this study assessed the MLC survivability under heat, acid, and bile stress. Moreover, the effects of microencapsulated and free LC addition to AVJD as a fortified product were determined. Physicochemical and sensorial properties of AVJD and the survival ability of LC were assessed during the storage period and GI condition.

## 2 MATERIALS AND METHODS

### 2.1 Materials

Erythromycin (Sigma), Anaerocult® C Merk, Gas Pack (Merck, Darmstadt, Germany), MRS broth (De Man et al., 1960), sodium citrate, peptone water, and MRS Agar were purchased from Merk company (Merck, Darmstadt, Germany). Sodium alginate was provided by Sigma Company (Sigma, Steinheim, Germany). The *Plantago major* seed was provided by Ahura Med, Marvdasht, Fars, Iran. It was collected from growing plants in Pasargad city, Fars province (South of Iran). Further plant identification was performed by the herbarium of the Fars Research Center for Agriculture and Natural Resources (FRCANR), Shiraz, Iran. A voucher specimen is deposited in the herbarium of the FRCANR, Shiraz, Iran.

### 2.2 Mucilage extraction

Deionized water was used to prepare water: seed ratio of 60:1 at pH 6.8 to extract *PMM* from whole seeds. The 0.1 M HCl or NaOH solution was used for adjusting pH in the water bath (75 ± 2.0°C). The water was preheated to a specific temperature before the seeds were added. The seed and water slurry were stirred with a motorized stirring paddle throughout the process (1.5 h). The seeds were passed through an extractor to isolate the mucilage from the expanded seeds, where a rotating plate scraped the mucilage from the surface of the seeds. The collected mucilage was then filtered, dried in an oven (overnight at 45°C), ground, and sieved through an 18 mesh sieve. The final dry powder is packaged and stored in a cool and dry place.

### 2.3 Bacterial cultivation

*The LC* (PTCC 1955) was obtained from the Persian Type Culture Collection. Among the *Lactobacilli*, LC is resistant to erythromycin. The *LC* was cultivated in selective MRS broth supplemented with 4 μg/mL erythromycin (Sigma) under microaerophilic and capnophilic conditions using Anaerocult® C Merk Gas Pack at 37°C for 48 h.

### 2.4 LR microencapsulation procedures

The *LC* broth culture was centrifuged at 2800 *g* for 10 min and then washed with 0.1% sterile peptone water (peptic digest of animal tissue and sodium chloride). The final volume of 5 mL was considered for the centrifuged washed cell. The estimated total bacteria count in the MRS broth was about 2.25 × 10^9^ CFU/mL. To perform the microencapsulation, an extrusion technique was applied. The 5 mL of centrifuged bacterial culture (9.3 × 10^11^ CFU/mL) was added to 15 mL of 1.5% sodium alginate solution. The bacterial suspensions were injected through a 0.11 mm needle into a sterile 0.1 mol/L CaCl_2_. It was subsequently placed in a refrigerator for 12 h before washing the microspheres with 0.1% peptone water and separated into (0.2, 0.4, 0.6, and 0.8% w/w) mucilage solution and gently shaken on the apparatus at 100 rpm for 40 min with orbital shaker (two‐stage method). The beads were then washed several times with 0.1% sterile peptone water. The morphology and aspect ratio of 20 microspheres were assessed by a digital microscope (Olympus BX51, Japan) and analyzed with Micromeasure software version 1.07. To calculate the aspect ratios, Equation (1) was finally applied:

(1) Aspect Ratio=MajorXaxis/MinorYaxis

The encapsulation yield (EY) was calculated and shown in Equation (2):

(2) $$\mathrm{EY}=\left(\log\;N/\log\;N0\right)\times 100,$$

where *N* is the number of viable cells (CFU/g of microsphere) after forming the microencapsulated cells and *N*0 is the number of viable cells (CFU/g of mixed alginate) taken initially to produce the beads (Pourakbar et al., 2023).

### 2.5 Scanning electron microscopy

The microspheres were subsequently vacuumed and examined with a scanning electron microscope (JSM‐5800LV, JEOL, Japan). Cross‐sections of lyophilized beads and AVJD were initially coated with gold. The outer surface and internal structure of the freeze‐dried beads were observed.

### 2.6 Color

Hunter (Lab) (Konica, Minolta, and CM‐5, Minolta, Osaka, Japan) was employed to evaluate the beads' color. The (L*) for darkness/lightness (0 black, 100 white), a* (− *a* greenness, ± *a* redness), and b* (− *b* blueness, ± *b* yellowness) color parameters were assessed.

### 2.7 Heat resistance of MLC and FLC

The heat resistance of FLC and MLC (8.75 to 9.28 log CFU/mL) was studied at 72°C (temperature degree for milk pasteurization) in MRS broth medium for 1, 2, 3, 4, and 5 min. After heat treatment, the samples were cooled with tap water. For releasing bacteria, sterile sodium citrate buffer 0.1 M (pH = 6.2) was used to finally collect the sample. The viability of the bacteria was determined by serial dilution and cultivation methods.

### 2.8 Survival in salt and acid stress

The glycine–HCl buffer (pH 1.5) containing 15% NaCl was chosen as salt and acid stress condition. The MLC and FLC were added to this medium. The pour‐plate technique in MRS selective medium was used to enumerate the effective cells in the samples at 0, 30, 60, and 90 min. The plates were finally incubated at 37°C for 48 h.

### 2.9 MLC and FLC survival during storage in MRS

The MLC and FLC were kept in MRS broth and stored at 4°C for 28 days, respectively. The viability of LC cells was enumerated in the 1, 7, 14, 21, and 28 days of storage time.

### 2.10 Preparation of probiotic AVJD

Control AVJD was prepared from 100 g of AVJD powder (Deraje Co, Yazd, Iran). The preparation was then boiled in 500 mL of water for 10 min and stirred with constant stirring. The endpoint of heating was determined by reaching a Brix value of 65^0^ using a refractometer. The mixture was then transferred to 75°C, poured into sterilized glass bottles, and allowed to stand. The FLC and MLC jellies were prepared using the same method. Equal final viable numbers (~9 log CFU/g) of bacteria in MLC and FLC forms were placed in AVJD and then poured into sterile glass containers. The physicochemical, sensory, and microbial analyses of all produced jellies were assessed during storage at 4°C.

### 2.11 Titratable acidity and pH

One gram of AVJD was taken and dissolved in 20 mL of distilled water in a conical flask, two to three drops of 2% phenolphthalein indicator were added, and then titrated with standardized 0.1 N NaOH till pink color appeared. The titratable acidity can be calculated as follows:

(3) $$\mathrm{Ta}=\frac{B\times 0.1\times 0.064\times 100}{W}$$

where Ta = titratable acidity; *B* = ml of 0.1 N NaOH; W = sample weight (Sikder and Ahmed 2019).

A digital pH meter was finally used to measure the pH of the control and probiotic AVJD (GPRT‐1400‐AN; Greisinger Electronic GmbH, Germany).

### 2.12 Survival of MLC and FLC in AVJD during the storage time

MRS agar was supplemented with 4 μg/mL erythromycin (Sigma). It was used to evaluate the FLC‐AVJD and MLC‐AVJD survivability during storage time. The AVJDs were stored at 4°C for 28 days, and LC count was carried out at 1, 7, 14, 21, and 28 days of storage. For the sampling of supplemented AVJDs, 1 and 0.1 mL of MLC‐AVJD and FLC‐AVJD were appropriately mixed with 9 and 0.9 mL of sterile sodium citrate 0.1M (pH = 6.2), respectively, and cultured in erythromycin MRS agar.

### 2.13 Survival of FLC and MLC exposed to simulated gastrointestinal condition

The method of Dokoohaki et al. (2019) was employed to analyze both simulated gastric fluid (SGF) and intestinal fluid (SIF). One and 0.1 g from AVJD containing FLC and MLC in 1 and 21 days of production were chosen for this assessment. The FLC and MLC were added to flasks containing SGF and incubated at 37°C in a shaker incubator (Shaker incubator, Labtron, Tehran, Iran), for 2 h (gastric phase). They were then added to SIF at pH 4.3–5.2. The LC counts were performed on triplicate samples after 30 min, 2, 4, and 6 h (each in three different flasks of the same experiment) using corresponding volumes (0.01 to 1 mL change). Aliquots of 0.01 and 0.1 mL of each preparation were immersed into erythromycin MRS agar before being incubated under microaerophilic conditions at 37°C for 72 h (Dokoohaki et al., 2019). The survival rate was calculated using Equation (4):

(4) $$\text{survival rate}\;\left(\%\right)=\frac{\mathrm{CFU}\;\mathrm{Log}\;N}{\mathrm{CFU}\;\mathrm{Log}N0}\times 100$$

*N*, the number of live probiotics (CFU/mL) after digestion and treatment of the simulated gastric and intestinal fluids and *N*0, the number of live probiotics (CFU/mL) before digestion and treatment in the simulated gastric and intestinal fluids.

### 2.14 Sensory evaluation of AVJD

Forty‐five trained panelists, who reported being consumers of AVJD products, were selected among students, faculty, and staff at Fars Agriculture Research and Education center, Iran, to evaluate each AVJD sample. The panel comprised men (38.5%) and women (61.5%) with an average age of 35–40 years. A 5‐point hedonic scale was applied to the AVJD sensory assessment. Panelists rated flavor, color, texture, and general acceptability. Compared to AVJD on the market, 5 means “very much like”, and 1 means “not at all” were applied for scoring. The panelists eventually assessed samples in the first and every 7 days during storage.

### 2.15 Texture of AVJD

Texture analyzer (Brookfield CT3 Texture Analyzer, 4500, USA) was used for TPA analysis of AVJD in the first and the last days of storage time. Using the TA11/1000 cylindrical probe, 40% of the AVJD cubes were compressed to twice their original height. The sample had a height of 30 mm and a diameter of 20 mm. A penetration depth of 20 mm was measured at the following speeds: 1 mm/s for the pretest, 1 mm/s for the test, and 10 mm/s for the posttest. Parameters such as hardness (g), cohesiveness, adhesiveness (mJ), chewiness (mJ), springiness (mm), and gumminess (g) were measured from the device. All these measurements were performed in at least three replicates at 25 ± 3°C for each sample.

### 2.16 Statistical analysis

Data were statistically analyzed using SPPS software (SPSS Ver 21.0). All statistical analyses were performed using one‐way ANOVA and Duncan's multiple range test (*p* < .05).

## 3 RESULTS AND DISCUSSION

### 3.1 Size, morphology, color, and encapsulation efficiency of beads

**FIGURE 1:**
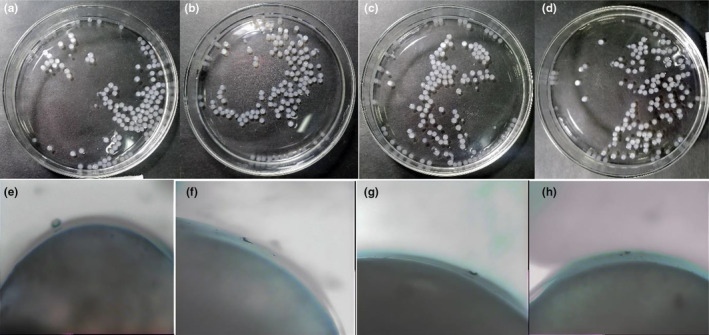
Microencapsulated *Lactobacillus curvatus* (MLC) from left to right, at concentrations of 0.2%, 0.4%, 0.6%, 0.8% (a–d) of *plantago major* mucilage (PMM), respectively (top row); and Light microscopy of MLC from left to right, at concentrations of 0.2%, 0.4%, 0.6%, 0.8% (e–h) of PMM, respectively used in microencapsulation (40×) (bottom row).

Macroscopic and microscopic images (Figure 1a–h) demonstrate the spherical particles (aspect ratio of 1.12) produced by the extrusion method. The spherical nature of the encapsulated beads prevented cell overgrowth within the encapsulated beads (Karimi et al., 2021). Similar results were reported by Castro‐Rosas et al. (2021) who produced alginate microspheres obtained by extrusion. Various factors affect the sphericity of microspheres. These include molecular weight of alginate, intrinsic viscosity, agitation speed, degree of polymerization, concentration of alginate, and air pressure applied to the system. There is a direct association between increasing agitation, higher pressure, and a greater chance of breakage of beads (Castro‐Rosas et al., 2021). Table 1 shows the average size of alginate PMM beads. The average of the alginate layer and PMM diameter was 3271.44 ± 47.8 to 3354.0 ± 154.44 μm and 35.33 ± 3.00 to 48.89 ± 1.03 μm, respectively. This average increases with the PMM concentration of the extrusion‐derived beads significantly (*p* < .05). In another research, the range size of beads was varied from 100 μm to 5 mm in diameter. This parameter affects the organoleptic properties of foods, and large microspheres can be disadvantageous when incorporated into food systems (Castro‐Rosas et al., 2021). It was also pointed out that the protective characteristic of encapsulation can be removed by reducing in diameter, whereas enhancing the capsule diameter effectively reduces the digestibility of pancreatic enzymes (Afzaal et al., 2019).

**TABLE 1:** Layers dimension and color parameters in microencapsulated *Lactobacillus curvatus.*

Bead	PMM%			
Parameters	0.2	0.4	0.6	0.8
Layers dimension (μm)				
Alginate layer	3271.44 ± 47.85a	3250.33 ± 67.11a	3325.56 ± 163.60a	3354.0 ± 154.44a
PMM layer	35.33 ± 3.00c	41.78 ± 1.86b	42.78 ± 9.51b	48.89 ± 1.03a
Color parameters				
L*	68.11 ± 8.71a	64.22 ± 4.66b	63.00 ± 6.42ab	61.22 ± 5.47b
a*	−1.89 ± 0.78a	−3.44 ± 0.88a	−3.64 ± 1.22a	−4.11 ± 0.93a
b*	15.00 ± 2.96c	21.22 ± 2.99b	27.00 ± 5.50a	28.44 ± 4.25a

*Note*: Data (mean ± standard deviation) are from three replications (*n* = 3). Means followed by different lowercase letters in row (*p* ≤ .05) by Duncan test.

The number of viable cells was 10.28 Log CFU/g in beads after forming the microencapsulated cells and 11.52 Log CFU/g in mixed alginate initially taken to produce the beads. So, the encapsulation yield was 89.23%. The formation of the alginate and oligosaccharides arabinogalactans combination may be one of the reasons for the high encapsulation yield. The presence of polysaccharides in the seed coat makes it gummy under warm and humid conditions (Noshad et al., 2021). This is thought to reduce the porosity of the bead's surface and prevent bacteria from escaping into the environment. Furthermore, encapsulation efficiency is directly associated with the variety of hydrogel matrices (Afzaal et al., 2020). For instance, it was reported that supplementing okra mucilage as a second layer in beads could increase the microencapsulation yield (90.14%) (Pourakbar et al., 2023).

### 3.2 Scanning electron microscopy

**FIGURE 2:**
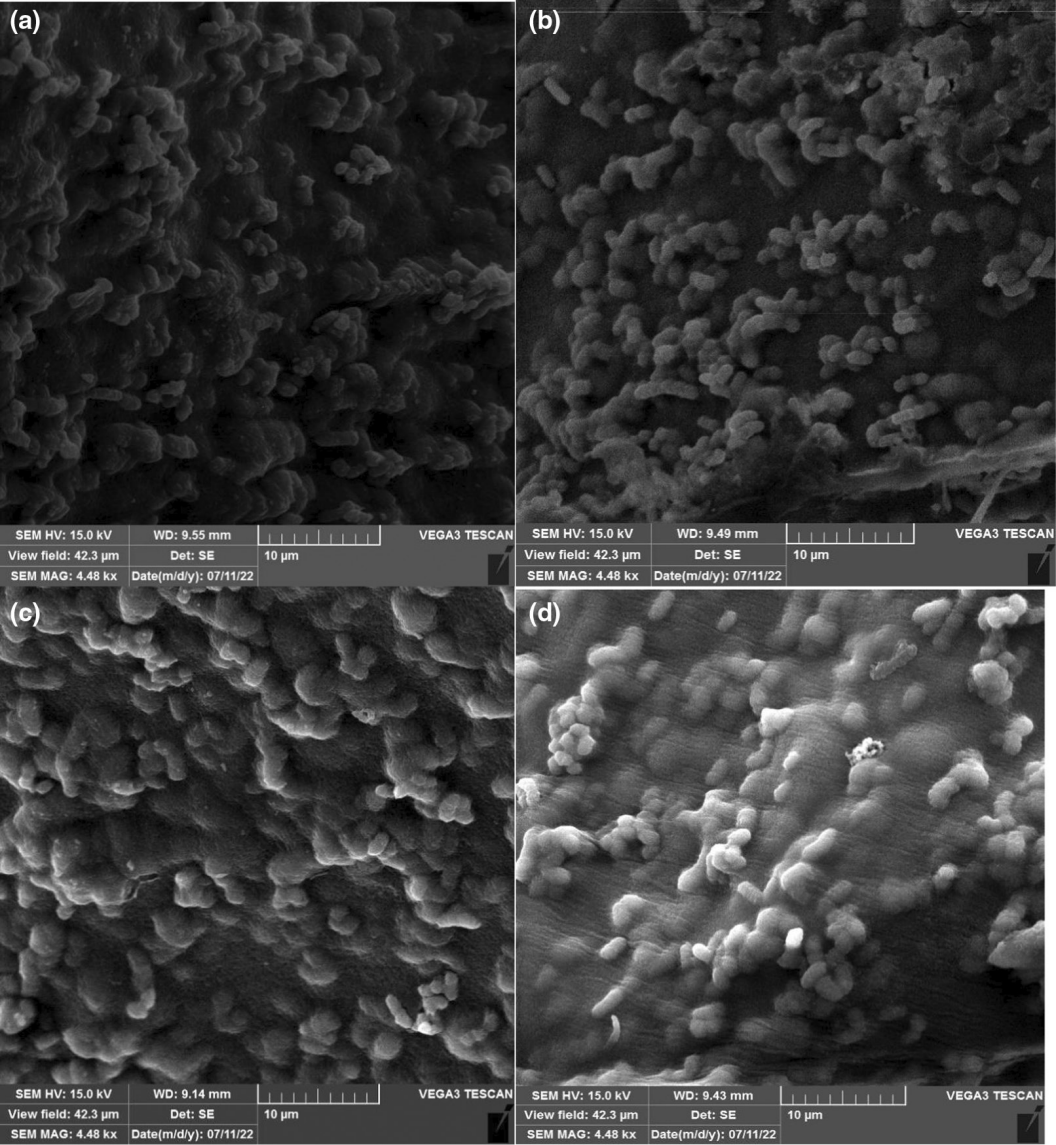
Scan electron microscopy of microencapsulated *Lactobacillus curvatus* (MLC) 0.2% (a), 0.4% (b), 0.6% (c), 0.8% (d) of *plantago major* mucilage, respectively, used in microencapsulation.

The shape of the microsphere was analyzed by scanning electron microscopy (SEM) (Figure 2a–d). The regular morphology was confirmed in the microscopic images. Pourjafar et al. (2020) reported a similar morphology for *L. acidophilus* and *L. rhamnosus* covered with a single‐layer coating (chitosan) and double (chitosan and Eu S100). A smooth appearance was noticed on the surface of the double‐coated capsules. The soft and homogenous shapes of the capsules resemble the findings of our study. An improved resistance to harsh conditions was remarkable by using this type of bead (Pourjafar et al., 2020). Moreover, numerous changes in the structural properties including surface characteristics, size, humidity, and configuration of microcapsules reported in previous studies. The rigidity of microcapsules is attributed to adding PMM which intensifies the hydrogel network to finally fill the remaining gaps (Hu et al., 2021). The uniform and smooth microstructure of the wall composition due to optimal proportion and adding 0.8% PMM was interesting. In addition, the distribution of *Lactobacillus* occurred in all samples. The penetration of bacterial cells into the matrix of multilayered microcapsules was confirmed by SEM images. Concomitant to increase the PMM concentration in the second layer of the bead, the smoother outer layer was observed.

### 3.3 Heat tolerance

**FIGURE 3:**
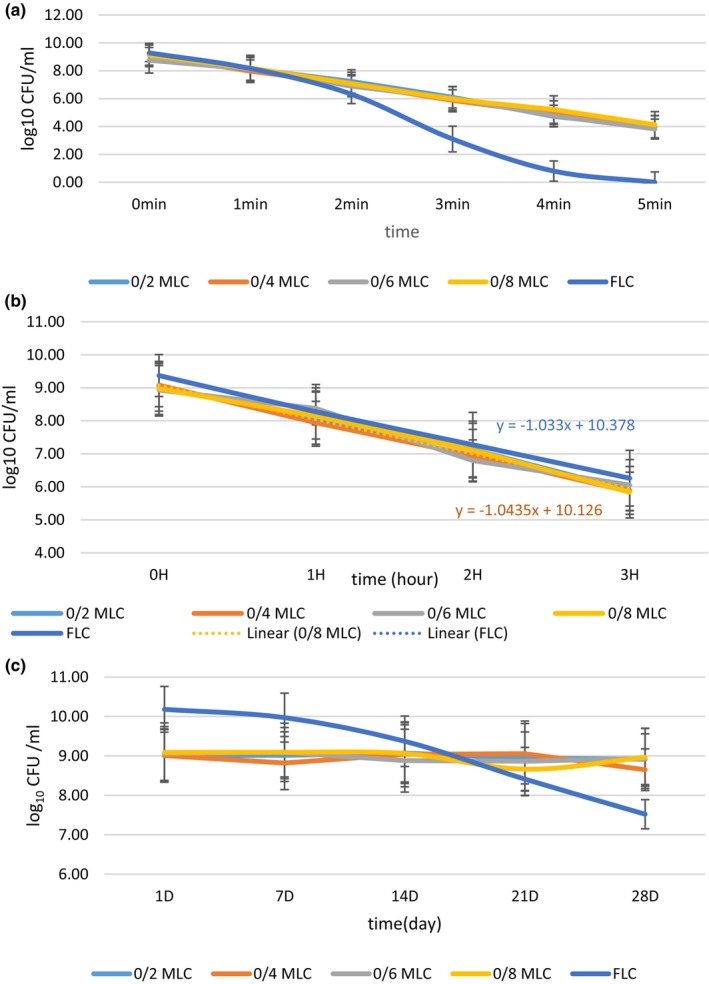
The survival of free *Lactobacillus curvatus* (FLC) and microencapsulated *Lactobacillus curvatus (MLC)* at concentrations of 0.2%, 0.4%, 0.6%, 0.8% at 72°C (a); acidic (pH 1.5) and salt (15% NaCl) (b) and during cold storage (c). Data (mean ± standard deviation) are from three replications.

We investigated the effect of temperature on FLC and MLC viability and tolerability (Figure 3a). As a result, MLC showed a higher survival rate (50.15%) than FLC (not detected within 5 min). Therefore, the temperature tolerance for MLC (72°C for 5 min) was higher than the free form. In similar research, Karimi et al. (2021) found that microencapsulated *Lactobacillus reuteri* had a survival rate (73.25%) after 5 min exposure to heat. The difference between our results and Karimi et al. (2021) studies may be related to the type of wall material and the number of bead layers (Karimi et al., 2021). The use of encapsulating bacteria increases thermal resistance because of a protective layer. Belyani et al. (2023) reported the effect of temperature changes on the stability of microencapsulated *Lactobacillus acidophilus La‐5* along different temperatures of 55–60°C. The encapsulating bacteria had a more resistance than the free one (Belyani et al., 2023). Particularly, an excellent survival ability to heat stress (72°C) was observed in the case of encapsulated *Lactobacillus acidophilus* with sodium alginate–soy protein isolate. The minimum level of *Lactobacillus acidophilus* (10^7^–10^8^ cfu/mL) was achieved, which is the advised therapeutic level in functional foods (Zeashan et al., 2020).

The temperature was a key factor influencing probiotic viability. The association of exposure time, temperature, and viability of encapsulated probiotics was shown in previous studies (Dokoohaki et al., 2019; Karimi et al., 2021). For example, the reduction of *Lactobacillus plantarum* in microencapsulated form (0.8 log CFU reduction) after heat treatment at 50°C for 5 min was fewer than the free form (5.7 log CFU reduction) (Ni et al., 2023).

### 3.4 Survival in acid and salt conditions

Figure 3b illustrates the viability of FLC and MLC in acid and salt solutions. The viability of both the FLC and MLC decreased with increased exposure time. At pH 1.5 and salt 15%, the FLC and MLC decreased viable number (~ 3.11 log CFU/mL) after 3 h of incubation. Verluyten et al. (2004) found that LC resists low salt concentrations (2%, wt/vol). However, high concentrations (6% wt/vol) of sodium chloride significantly inhibited LC growth. Primarily, NaCl exhibited an inhibiting role due to acting as a water activity (aw) lowering agent (Verluyten et al., 2004). In addition, high degrees of acid resistance are presented by the LC. It usually possesses acid resistance mechanisms like F1F0‐ATPase, glutamate decarboxylases, or arginine deiminase (ADI) systems, which protect it against acid (Janßen et al., 2020). Therefore, the decrease in the number of bacteria may be related to the sensitivity of these bacteria to salt in the environment (Luan et al., 2022).

### 3.5 MLC and FLC survival during storage in MRS

Details of MLC and FLC survival are shown in Figure 3c. Following 28 days of storage, a substantial reduction in the viable cell count (~ 2.66 log CFU reduction) was seen in FLC. The MLC count was nearly constant during storage time. It was previously reported that the number of viable probiotics in microencapsulated samples was higher than in the free form during storage (Ni et al., 2023). The encapsulated *Lactobacillus plantarum* with alginate–gelatine gel viable number decreased by 2.1 log CFU/mL during storage. In contrast, the free probiotic had more reduction in viable number after 6 days of storage (1.51 log CFU/mL) (Ni et al., 2023). This result was in contrast with our result. One of the reasons may be related to the time of sampling and storage medium. The storage time in their research was shorter than mine. Moreover, the storage medium in their research was normal saline, while in our research, it was MRS broth. Therefore, enrichment media could extend probiotic viability during storage time (Ni et al., 2023).

### 3.6 Titratable acidity and pH

**FIGURE 4:**
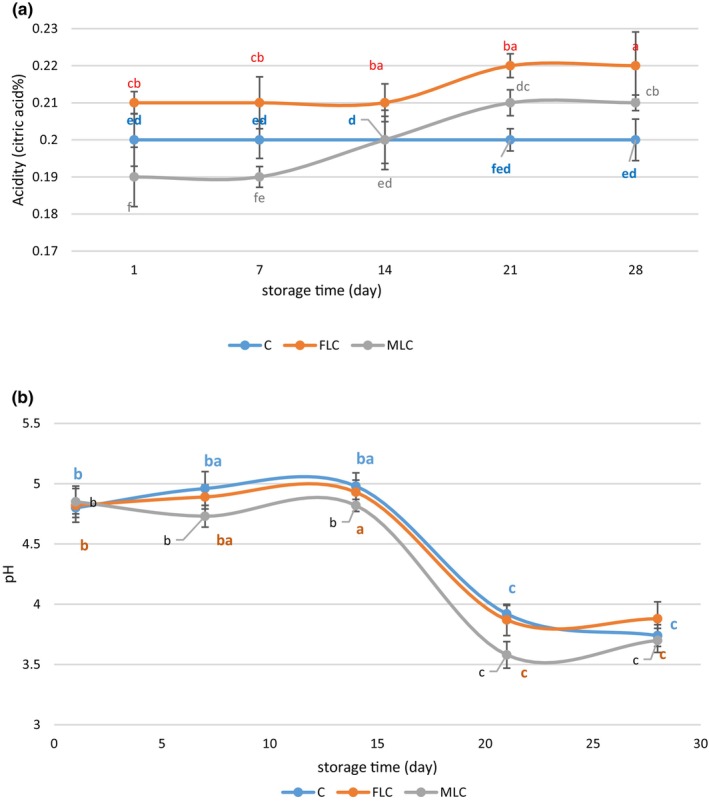
Acidity(a) and pH (b) values of free *Lactobacillus curvatus* (FLC) and microencapsulated *Lactobacillus curvatus* (MLC) and control (C) AVJD during storage time at 4°C in AVJD. Data (mean ± standard deviation) are from three replications.

The content of acidity and pH are shown in Figure 4a,b. Total acidity and pH are the main characteristics used to evaluate the AVJD that help in the desirable balance of sugary acids necessary for a pleasant taste (Curi et al., 2023). While the pH had no significant difference among the samples at the end of storage (*p* > .05), the acidity of MLC and FLC was significantly more significant than the C sample (*p* ≤ .05). In the C sample, a significant decrease in pH during storage time was observed (*p* ≤ .05). The remaining microorganisms in the AVJD, which are not damaged during the production process, are one of the reasons for these changes. However, pH and acidity are not consistently or poorly correlated in certain foods (Paulson & Stevens 1974). The higher acidification at the end of storage is probably associated with a unique fermentation of LC. However, this did not adversely affect the product (Funck et al., 2019). High acidity in the ice cream containing free and encapsulated bacteria was seen. It can be related to the production of organic acid by probiotic bacteria (Maleki et al., 2023). Based on the obtained results, the acidity of FLC had a more significant value (*p* ≤ .05) than MLC because of the decreasing activity of MLC. The size of the alginate layer is one of the aspects that act on the metabolic activity of microencapsulated bacteria in products. The more layers of encapsulation led to less acid released. In this study, we used two layers as a wall for encapsulation. Furthermore, the layers formed around the bacteria may slow food intake, reducing the proportion of organic acids (Ghasemnezhad et al., 2017). A similar result was found by the other researchers (Saniani et al., 2023).

### 3.7 Survival of MLC and FLC in AVJD during the storage time

**FIGURE 5:**
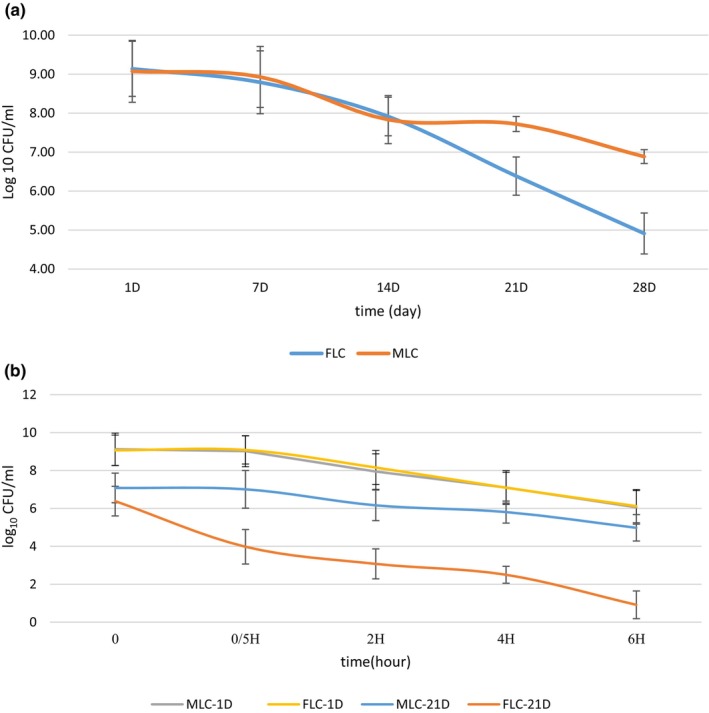
The free *Lactobacillus curvatus* (FLC) and microencapsulated *Lactobacillus curvatus* (MLC) in AVJD during storage (a) and in simulated gastrointestinal condition (b). Data (mean ± standard deviation) are from three replications.

Cell damage and loss of viability can occur throughout food processing and storage. A successful encapsulation process should therefore ensure the viability of the probiotics through the various stages of processing and maintain their activity during storage (Neuenfeldt et al., 2022). In comparison with FLC, the MLC survival was more significant than FLC during the storage stability test at 4°C. An increase in the storage stability of MLC was observed (Figure 5a). During the storage time, the survival rate of all supplemented AVJD began to decline. There was a difference between the FLC and the MLC count in the 28 days of storage time. FLC had a marked decrease in viable counts during 28 days of storage, dropping from 8.93 log CFU/mL to 4.91 log CFU/mL, with a viability percentage of 54.98%. However, the reduction of MLC was 2.19 log CFU/mL, and the survival rate was 77.85% during storage. Similarly, Li et al. (2023) used protein‐isolated fibrils, sodium alginate, carboxymethylcellulose, and xanthan gum to protect Lactobacillus plantarum. The data showed that the count of free cells reduced from 8.67 log CFU/mL to 1.27 log CFU/mL with a viability of 14.68% after 60 days of storage at 4°C. However, the survival rate of encapsulated bacteria was 60% (Li et al., 2023). It differs from the current research results. One of the reasons for this difference is due to the types of bacteria and wall materials. Encapsulation methods can improve the stability of probiotics during storage. This process is suggested as an effective procedure to protect probiotics during storage. Various factors such as the extent of metabolization, pH reduction, and treatment type are influencing the viability of probiotic bacteria to recommend the use of prebiotics in a formulation (Shahraki et al., 2023).

### 3.8 Survival in simulation gastrointestinal condition

Figure 5b shows the survival of FLC and MLC under GI conditions over time. The cell viability decreased, and the survival rate reached 14.24% and 71.04% for FLC and MLC samples, respectively. PMM showed a protective effect as a proper physical barrier. The alginate bead surface had a porous structure. The biopolymer network formed in PMM–alginate may be responsible for the slow diffusion of GI fluid through the wall material and decreased outflow of the entrapped probiotics from the microcapsules to the exterior environment of the gastrointestinal tract (Parsana et al., 2023). The viability of probiotics under harsh conditions of upper gastrointestinal tract is affected by the pore size of alginate‐based microcapsules. The recommended pore size of the microcapsule is smaller than the size of hydrogen ions (<1 nm) and enzymes (<5 nm) (Khorshidi et al., 2021).

The results of this research are supported by the study of Shafizadeh et al. (2020). In their study, flaxseed mucilage was used for co‐encapsulating probiotic *L. casei* in the alginate (ALG) matrix. The viability of microencapsulated bacteria was higher than that of free cells under GI conditions (Shafizadeh et al., 2020).

### 3.9 Sensory evaluation

**FIGURE 6:**
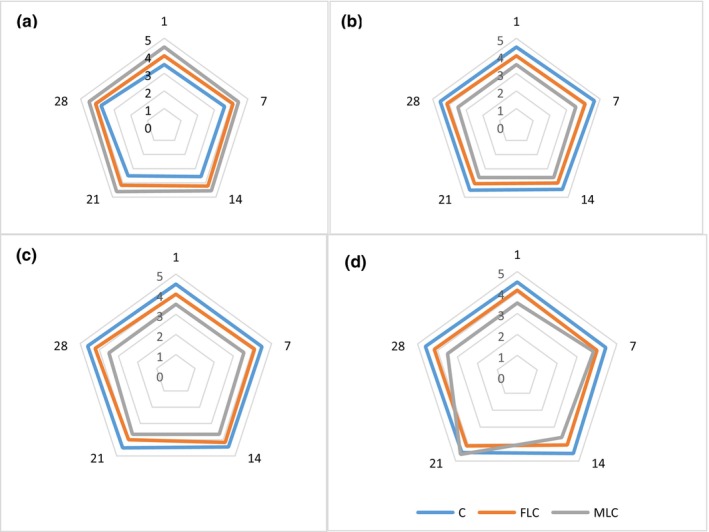
Sensory properties of control (c); microencapsulated *Lactobacillus curvatus* (MLC), free *Lactobacillus curvatus* (FLC) during storage time in AVJD. Sensory parameters consist of Texture (a); Color (b); Flavor (c); Acceptability (d).

The sensory properties of AVJD were evaluated and are shown in Figure 6a–d. MLC showed the highest textural values probably due to the release of wall material (PMM) in AVJD. The color and acceptability scores were at least in MLC, which can be associated with the development of grainy texture because of MLC wall materials. Moreover, PMM and alginate provide a highly viscous medium to enhance MLC texture. According to another report and in confirming the result of this research, sensory evaluation demonstrated that judges perceived the presence of beads in probiotic food sensed (Saniani et al., 2023).

### 3.10 Texture of AVJD

The assessment of texture parameters showed that with increasing storage time, hardness increased significantly (*p* ≤ .05) in all samples (Table 2). Among the samples, MLC had the highest hardness significantly (*p* ≤ .05) at the 28th of the storage period. However, significant changes in structural parameters were expected due to modification of the AVJD protein crosslinking pattern. It seems that the incorporated beads make the gel more rigid, probably because the amount of water absorbent in the network decreases and the solid structure (beads) increases. An increase in hardness (1.95–6.80) N was also seen in gelatin desserts with microencapsulated *Lactobacillus fermentum* when stored at 4°C for 25 days (Amani et al., 2017; Dokoohaki et al., 2019). The result of this research fits with the report of Didar (2023) who attributed the higher hardness of enriched white chocolate with co‐encapsulated *Lactobacillus acidophilus* (La‐5) (Didar, 2023).

**TABLE 2:** Texture parameters in C, MLC, and FLC jellies during 28 days of storage at 4°C.

Samples	Day	Hardness (g)	Adhesiveness	Cohesiveness (mJ)	Springiness (mm)	Gumminess (g)	Chewiness (mJ)
C	1	245.60 ± 8.16d	0.08 ± 0.03b	1.06 ± 0.03b	20.23 ± 0.23c	193.67 ± 5.13c	38.80 ± 0.30c
	28	627.0 ± 11.53b	1.20 ± 0.05d	0.31 ± 0.04d	20.30 ± 0.21c	254.53 ± 5. 76b	50.18 ± 3.97b
FLC	1	114.0 ± 5.31f	0.04 ± 0.01a	1.52 ± 0.11a	23.86 ± 1.08b	89.85 ± 3.67d	21.81 ± 1.20d
	28	212.0 ± 6.56e	0.16 ± 0.02c	0.85 ± 0.04c	24.95 ± 1.16b	182.67 ± 4.62d	25.34 ± 1.41d
MLC	1	523.0 ± 6.0c	0.58 ± 0.03c	0.83 ± 0.04c	18.36 ± 0.41d	82.93 ± 2.44d	20.25 ± 0.66d
	28	911.33 ± 10.07a	0.95 ± 0.05e	0.17 ± 0.02e	26.50 ± 0.79a	763.47 ± 23.99a	134.13 ± 5.61a

*Note*: Data (mean ± standard deviation) are from three replications. Means followed by different lowercase letters in column differ (*p* < .05) by Duncan test.

Abbreviations: C, control; FLC, free *Lactobacillus curvatus*; MLC, microencapsulated *Lactobacillus curvatus*.

Adhesiveness (also known as stickiness) is determined by the action necessary to overcome the attractive forces between the surface of the food and the body part that comes into contact (e.g., tongue, teeth, palate). Adhesiveness increased significantly during the storage period in all samples (*p* ≤ .05). Among the samples, the C sample had the highest significant adhesiveness in the 28th of storage time (*p* ≤ .05). As an essential parameter, hardness and adhesiveness are positively correlated with microcapsule mechanical strength. Adhesiveness value is related to surface properties and the combination effects of adhesiveness and cohesiveness forces. Moreover, they are associated with the molecular structure of the product. Studies have shown that stronger jellies have higher adhesive strength value (Yusof et al., 2019). In this research, adding FLC and MLC to AVJD raised the adhesiveness significantly (*p* ≤ .05). Such increased adhesiveness of condensed milk‐based desserts was also reported (Aragon‐Alegro et al., 2007).

Cohesiveness is described as a large number of broken protein connections during stressful circumstances and springiness (the ability to recover its initial condition after applying a deformation) (Sekhavatizadeh et al., 2022). Cohesiveness decreased significantly (*p* ≤ .05) with increasing storage time. Among the samples, the most significant value (*p* ≤ .05) of cohesiveness was related to FLC at the end of storage time.

**FIGURE 7:**
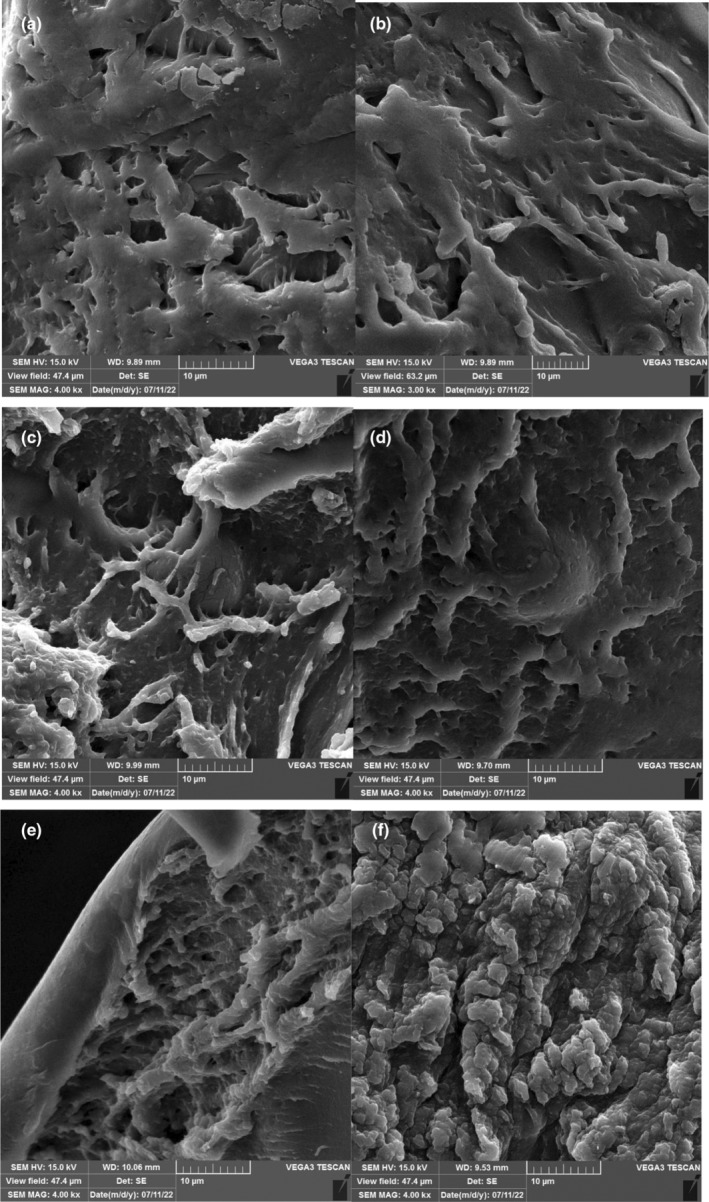
The SEM image of AVJD contains microencapsulated *Lactobacillus curvatus* (MLC), free *Lactobacillus curvatus* (FLC), and Control (c), during storage time. C in 1st (a); FLC in 1st (b); MLC in 1st (c); C in 28th (d); FLC in 28th (e); MLC in 28th (f) of storage time.

Springiness is the speed and extent of deformed material recovery to its initial condition after removing a specific force. In our study, springiness increased significantly (*p* ≤ .05) in all samples except C‐AVJD during storage time. The MLC had the most significant springiness value among the samples in the 28th of storage (*p* ≤ .05). The particle diameter could affect the microstructural and textural properties of food, like springiness (Kouamé et al., 2023). For instance, the large protein particles in MLC‐AVJD may increase springiness (Figure 7). The springiness increased during the storage time. Consistent with current results, a previous study showed springiness increased after a more extended incubation period (Mudgil et al., 2017).

Gumminess is closely connected with the statutes of hardness and cohesiveness. The MLC revealed the most significant (*p* ≤ .05) value in gumminess, and this parameter increased during the storage time in all samples. In similar research, Sekhavatizadeh et al. (2023) found that increased fortified samples during storage resulted from the gumminess, which was also consistent with our study. Sah et al. (2016) found that high gumminess was created by the pH reduction, structural shrinking, and rearrangement of the protein structure in the product (Sah et al., 2016; Sekhavatizadeh et al., 2023).

The chews required for a specific amount of food to decrease swallowing steadiness are rationally present by the term chewiness (Borhanpour et al., 2021). Moreover, the chewiness presented the same behavior as the gel hardness. The chewiness value was the most significant (*p* ≤ .05) in MLC. This parameter increased during storage time in all samples.

### 3.11 SEM image of AVJD

To investigate changes in microstructural properties between C, MLC, and FLC during storage, SEM was used. The images were attained from the C, MLC, and FLC‐AVJD samples on the first (Figure 7a–c) and 28 days (Figure 7d–f) of storage. On day 1, the protein network of CAVJD (Figure 7a) was more open, less dense, and contained more cavities. However, the FLC and MLC‐AVJD samples had less space and a denser structure. A similar study showed that a gel‐like structure was observed in microencapsulated yogurt on the first day of storage, which is believed to be due to dense protein networks (Pourakbar et al., 2023). The MLC structure showed globular protein aggregates, presumably due to PMM acting as protein binders. The PMM absorbed water, expanded, and filled the graves. Therefore, the open structure of the protein micelle network was a consequence of some of the solubilizing molecules within the PMM that could enter the AVJD protein (Sandoval‐Castilla et al., 2004).

## 4 CONCLUSIONS

The extrusion method was successfully employed to encapsulate the LC in the alginate‐PMM beads. Survival of encapsulated LC in PMM and alginate was improved survival ability compared to the free cells at low pH (pH 1.5) plus 15% saline, heat stress at 72°C, and long‐term storage (28 days). Compared with quince seed mucilage, the MLC containing PMM showed better survivability during storage time. In addition, the MLC plus PMM revealed a greater chance of survival at low pH (pH 1.5) and 15% saline, heat stress at 72°C, and long‐term storage (28 days) compared to *Pistacia atlantica* subsp. *Kurdica* gum (Dokoohaki et al., 2019; Sekhavatizadeh et al., 2021). Encapsulated LC can survive in simulated gastrointestinal fluid and AVJD during storage. Encapsulation has proven to be an excellent method of protecting probiotics in the gastrointestinal environment and under harsh conditions. Alginate‐PMM beads show potential as new carriers for oral delivery of LC. Moreover, these wall materials can increase the survivability of LC against low pH, salt, heat, and storage when compared to other materials. One of the limitations of bead usage in food is the large size of the beads (3000 μm), which reduces sensory score among the panelists. Moreover, this method can be complex for large‐scale production because of the slow formation of beads. The suitable wall materials with excellent encapsulation efficiency would be interesting to determine in future work. Besides, the production of smaller beads with appropriate sensory properties would be interesting to determine in future studies.

## AUTHOR CONTRIBUTIONS

**Seyed Saeed Sekhavatizadeh:** Conceptualization (lead); writing – original draft (lead). **Maryam Derakhshan:** Investigation (equal). **Mohammad Ganje:** Data curation (lead); methodology (lead). **Saeid Hosseinzadeh:** Writing – review and editing (lead).

## FUNDING INFORMATION

This research did not receive any specific grant from funding agencies in the public, commercial, or not‐for‐profit sectors.

## CONFLICT OF INTEREST STATEMENT

The authors declare that they do not have any conflict of interest.

## ETHICS STATEMENT

This study does not involve any human or animal testing.

## Data Availability

Data will be made available on reasonable request.
